# A photoacoustics-enhanced drilling probe for radiation-free pedicle screw implantation in spinal surgery

**DOI:** 10.3389/fbioe.2022.1000950

**Published:** 2022-09-15

**Authors:** Li Liu, Yongjian Zhao, Ang Li, Xianghu Yu, Xiao Xiao, Siyu Liu, Max Q.-H. Meng

**Affiliations:** ^1^ Department of Electronic Engineering, The Chinese University of Hong Kong, Hong Kong SAR, China; ^2^ Department of Electronic and Electrical Engineering, Southern University of Science and Technology, Shenzhen, China; ^3^ School of Science, Nanjing University of Science and Technology, Nanjing, China

**Keywords:** multi-scale navigation and sensing, ultrasound-based surgical navigation, photoacoustic endoscopy, robotic-assisted spinal surgery, cancellous bone characterization, *in situ* tissue sensing, pedicle screw implantation

## Abstract

This article proposes a novel intra-operative navigation and sensing system that optimizes the functional accuracy of spinal pedicle screw implantation. It does so by incorporating radiation-free and multi-scale macroscopic 3D ultrasound (US) imaging and local tissue-awareness from *in situ* photoacoustic (PA) sensing at a clinically relevant mesoscopic scale. More specifically, 3D US imaging is employed for online status updates of spinal segment posture to determine the appropriate entry point and coarse drilling path once non-negligible or relative patient motion occurs between inter-vertebral segments in the intra-operative phase. Furthermore, a sophisticated sensor-enhanced drilling probe has been developed to facilitate fine-grained local navigation that integrates a PA endoscopic imaging component for *in situ* tissue sensing. The PA signals from a sideways direction to differentiate cancellous bone from harder cortical bone, or to indicate weakened osteoporotic bone within the vertebrae. In so doing it prevents cortical breaches, strengthens implant stability, and mitigates iatrogenic injuries of the neighboring artery and nerves. To optimize this PA-enhanced endoscopic probe design, the light absorption spectrum of cortical bone and cancellous bone are measured *in vitro*, and the associated PA signals are characterized. Ultimately, a pilot study is performed on an *ex vivo* bovine spine to validate our developed multi-scale navigation and sensing system. The experimental results demonstrate the clinical feasibility, and hence the great potential, for functionally accurate screw implantation in complex spinal stabilization interventions.

## Introduction

The population is aging rapidly worldwide. Alongside population aging, the incidence of spinal degenerative pathologies (e.g., herniated discs, narrow lumbar canals) and/or vertebral osteoporotic fractures has been increasing, imposing a high socio-economic burden on eldercare and healthcare systems (Compston et al., 2019; Wang et al., 2020). Once such spine-related diseases occur, the vertebrae may no longer function properly, and, if unattended, become harmful to the surrounding nerves and tissues(Chapman, 2019). It is commonly recognized that spinal fusion surgery, which involves pedicle screw implantation to stabilize the spine, is effective in the recovery of spinal function (Germon and Hobart, 2015). During the procedure, the screws need to pierce the pedicles of the vertebrae and enter the vertebral body. Afterward, the implanted screws are attached to rods that stabilize the affected segments. Such surgery is highly challenging and technically demanding (Maria et al., 2020). First, the pedicle region is so small (around a few millimeters), it is extremely difficult to determine an accurate and safe trajectory for the drilling hole. Since the volume of the pedicle screw is relative to that of the pedicle, the margin of error needs to be within one millimeter to prevent screw misplacement or further cortical wall perforation (Lopera et al., 2010). Moreover, the lack of real-time feedback in the intra-operative phase means the success of pedicle screw implantation largely depends on subjective experience of the surgeon. To summarize, the proximity of vital neural and vascular structures, the added variability of patients, and the region-dependent morphology of the vertebrae, all contribute significantly to the difficulties with accurate screw implantation (Lonstein et al., 1999; Yamada et al., 2022). Several advanced surgical aids have previously been introduced to mitigate the associated complications and to improve accuracy. These are generally divided into two categories: global spatial navigation and local tissue sensing techniques.

The global spatial navigation technique mainly involves computer or robotics-assisted spinal surgery, navigated by macroscopic imaging modalities (Helm et al., 2015; Ahern et al., 2020). Since spinal navigation systems allow for either intra-operative imaging or pre-operative imaging with intra-operative updates, conventional navigation paradigms include intra-operative 2D fluoroscopic navigation (Foley et al., 2001), pre-operative CT with manual intra-operative registration (Nottmeier and Crosby, 2007), pre-operative CT with fluoroscopic registration and update, and intra-operative CT or CBCT (Tian et al., 2011; Dea et al., 2016; Cool et al., 2021; Zhang et al., 2021; Felix et al., 2022; Tabarestani et al., 2022). Common to these solutions is their association with X-ray-like imaging (fluoroscopy or CT-based navigation solutions featuring ionizing radiation), which can harm both patient and surgeon (Hecht et al., 2018; Ahern et al., 2020).

The other category of intelligent surgical aids is associated with local tissue sensing, also referred to as sensor-enhanced surgical instruments, that identify different tissue types and possess an integrated warning component. Intensive studies have been conducted into local tissue sensing instruments for spine-related surgeries. Li et al. (2022) proposed a surgical status perception method using an installed acceleration sensor and force sensor in robot-assisted spine surgery that mimics the surgeon’s tactile sensing. Experiments have proved that the fusion of two tactile signals can significantly improve status recognition accuracy. Electromyography is another option, although not widely adopted due to its lower sensitivity (Duffy et al., 2010). Furthermore, other professionals are required to interpret these signals, for the technology fails to predict an impending cortical bone breach in advance. To date, the only commercially available product, the so-called Pedi-Guard probe instrument, is based on electrical conductivity measurement to indicate proximity to the cortical wall of the vertebra (Ovadia et al., 2011). It can distinguish different tissues by comparing electrical conductivity. However, it lacks directional information about the sensed signals (Bolger et al., 2007). In other words, when the instrument tip is close, but parallel to the cortical wall, the sensing technique issues a warning. Yet even if it correctly detects an impending cortical breach, the surgeon still has no idea in which direction the breach is about to occur, because the electrical conductivity measurement occurs in all directions simultaneously. This means that when the instrument issues a warning, the surgeon must make the next attempt without any directional input.

Endoscopic ultrasound (EUS) also holds the potential for *in situ* tissue sensing and characterization (Mujagic et al., 2008; Manbachi et al., 2014). However, experimental results have proven unreliable due to the high acoustic impedance to the US from the bone (Aly et al., 2011). For superior image quality and tissue composition imaging characteristics, photoacoustic imaging (PAI) has been introduced as an alternative (Steinberg et al., 2019; Gonzalez et al., 2021). First discovered in 1880 by Bell, the PA effect has developed as an emerging medical imaging modality, and is widely adopted in various fields (Zhou et al., 2014). The principle of PAI is as follows: once the tissue is exposed to pulsed laser beams, it absorbs the energy and converts it into heat. Simultaneously, its temperature increases locally and thermal expansion occurs (Xu and Wang, 2006). Such expansion enables the tissue to emit ultrasonic waves, which are detected by an ultrasound transducer for subsequent image reconstruction and visualization (Kruizinga et al., 2014). PAI combines the merits of both optical and ultrasound imaging, yielding high-quality images with high resolution and contrast (Xu and Wang, 2006). Furthermore, it is radiation-free, and capable of both morphological and functional imaging. It thus holds huge potential for clinical translation (Beard, 2011). According to differences in excitation source and image reconstruction techniques, PAI can be categorized into different types. One of these is photoacoustic tomography (PAT), which exploits an unfocused beam for imaging (Sangha and Goergen, 2016). In general, there are several transducers, and the collected information is transferred to the computer for image reconstruction. Another type is photoacoustic microscopy (PAM), which employs a focused laser and transducer without needing a complex reconstruction algorithm (Yao and Wang, 2013). Unlike PAT, PAM has the advantage of imaging precise tissue samples and cells. The last type is photoacoustic endoscopy (PAE), a modality usually applied in invasive operations, which has a high standard in relation to the size of the probe (Joon-Mo Yang et al., 2009; Guo et al., 2020). In sum, PAI can generate images of high quality across a broad range of applications and holds huge potential in tissue sensing and characterization (Zhao et al., 2019). More recently, Gonzalez et al. (2021)developed a combined US and PAT guidance system aimed at avoiding the pedicle screw misplacement and accidental bone breaches that can lead to nerve damage. Pedicle cannulation was conducted on a human cadaver, with co-registered PAT and US images acquired at various time points during the procedure. However, the US/PAT guidance system only focuses on macroscopic anatomical navigation, without taking fine-grained local tissue characterization into consideration. To the best of our knowledge, local bone tissue sensing inside the vertebrae, based on a PAE-enhanced surgical instrument for guiding pedicle screw implantation, has not previously been studied.

In this article, we propose for the first time, a multi-scale surgical navigation and sensing system for pedicle screw insertion based on radiation-free dual-mode US/PA. More specifically, a macroscopic US imaging system achieves contextual awareness for global guidance of the drilling entry and coarse insertion path, while a mesoscopic PAE-enhanced smart probe instrument offers *in situ* tissue characterization for fine path optimization of screw insertion. Hence, in combination with US image guidance, such PAE sensing is exploited to monitor and even warn of cortical bone breaches inside the vertebrae, thereby optimizing screw implantation in complex spine-related surgeries.

## Materials and methods

### Radiation-free, multi-scale navigation and sensing systems for pedicle screw implantation optimization

**FIGURE 1:**
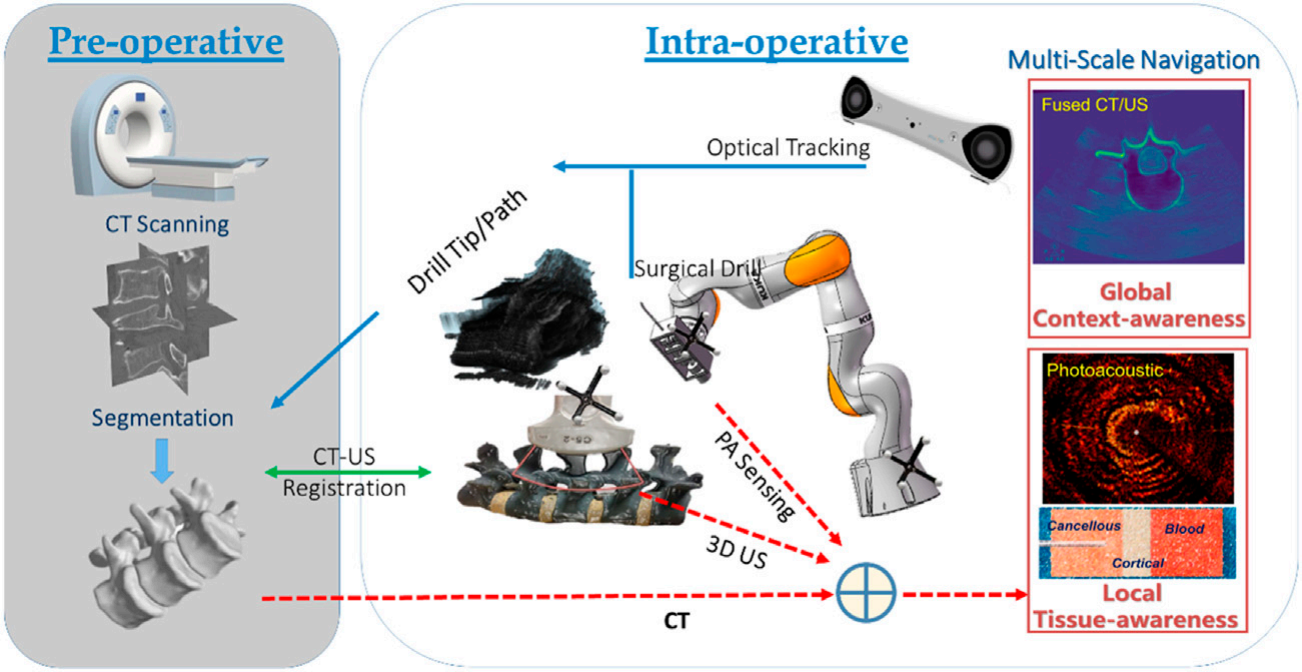
Multi-scale surgical navigation and sensing paradigm based on radiation-free intra-operative US/PA imaging.

The overall system configuration of the multi-scale surgical navigation and sensing paradigm, based on radiation-free intra-operative US/PA imaging, is presented in Figure 1:

The combination of global context-awareness via pre-operative CT and intra-operative US, along with local tissue-awareness via *in situ* PA sensing at a clinically relevant mesoscopic scale can enhance comprehensive perception for complication-free guidance of bone drill and pedicle screws through vertebrae. Specifically, a CT scan is performed and subsequently segmented to produce a 3D model of the bony surface of the spine. Once non-negligible patient movement occurs during the operation, the tracked US probe is scanned to obtain a series of 2D US images, yielding 3D volumetric data of the affected vertebral segments. In the registration phase, US-CT registration is conducted via automatic bony surface detection in US images, followed by Iterative Closest Point (ICP)-based point cloud registration. Afterward, the data on the drill bit, and the updated insertion path—due to any spinal segment posture variation—can be overlaid on a fused CT/US volume. Thus, with the position tracking data of the drill, the multi-scale navigation and sensing system allows for both context-aware global navigation and tissue-aware local sensing in complex spinal surgeries: 1) *Context-aware global navigation* visualizes the drill bit and insertion path from the fused CT model/US volume of the affected spine in real time. Thereafter, navigation of the drill and screw implantation, associated with an online updated insertion path, is effected based on the posture tracking data and the related US-CT registration result. 2) An embedded PAE probe inside the surgical drill is simultaneously employed for *in situ* identification of the tissue types aside of the drill bit (e.g., cortical and cancellous bones, and critical vascular/neurological structures) for optimizing fine orientation of the drilling probe in *tissue-aware local sensing*. Hence, the comprehensive fused perception serves as a multi-scale navigation and sensing paradigm enabling accurate screw implantation in complex spine-stabilization interventions.

### Macroscopic 3D ultrasound-guided global contextual navigation

#### 3D US imaging system setup

**FIGURE 2:**
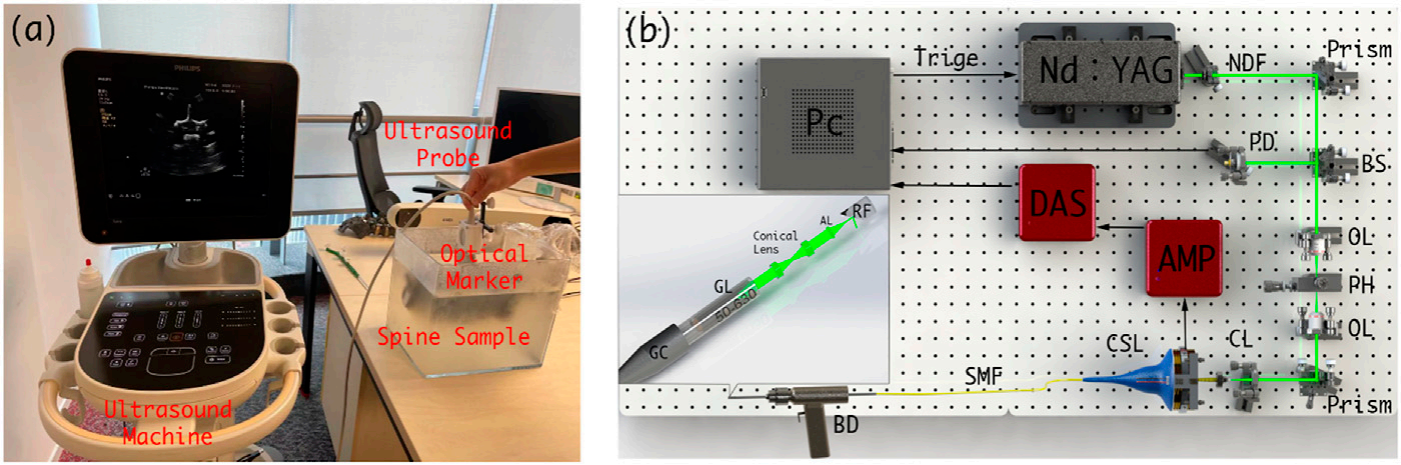
**(A)** Schematic diagram of the intra-operative US navigation system. **(B)** Schematic diagram of drilling probe. PC: personal computer, NDF: Neutral Density Filter, PD: Photodiode, BS: beam split, OL: object lens, PH: pin hole, CL: collimator, CSL: conductive slip, SMF: single model fiber, BD: bone drill, GC: grin collimator, GL: Grin Lens, AL: aspherical lens, RF: reflector.

**FIGURE 3:**
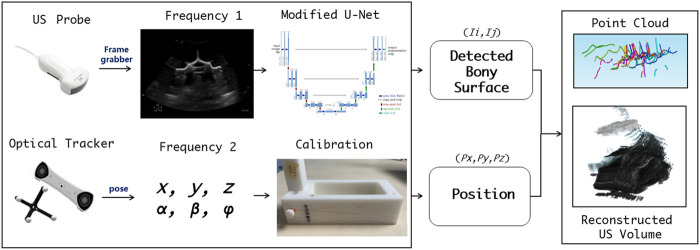
Workflow for acquisition, calibration, segmentation, compounding, and visualization of the US volume.

Prior to spinal surgery, a patient usually receives routine CT scanning for pre-operative examination and diagnosis. Afterward, challenging manipulations, such as pedicle screw implantations, are executed with the assistance of image-based surgical navigation in the intra-operative phase. Relative motion and deformation between the inter-vertebral segments can occur due to interactive force between the drill and the affected vertebrae. Therefore, to achieve on-the-fly status updates during the operation, a 3D US imaging system is established for continuous monitoring of the spinal segment being operated upon. Figure 2A and Figure 3 demonstrate the acquisition, calibration, segmentation, compounding, and visualization of the US volume. Afterward, such reconstructed US volume can be used intra-operatively in conjunction with the spinal bone surface model segmented from the pre-operative CT, together providing a global contextual perception for accurate pedicle screw implantation.

#### Pre-operative CT registration with intra-operative 3D US

To achieve co-registration between the reconstructed US volume and the bone surface model segmented from CT, two consecutive tasks are performed, i.e., bone surface detection of US images, followed by hierarchical coarse-fine point cloud registration.

**FIGURE 4:**
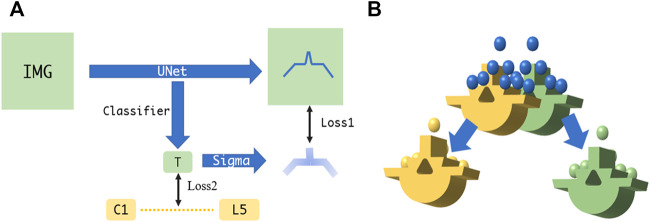
**(A)** Augmented U-Net using two losses for accurate bone surface detection. **(B)** Fine point cloud registration between US and CT.

Due to the physical nature of spinal sonography for osseous tissue imaging, only bone surface can be visualized on US images, which are highly sparse, thus making it difficult to learn via a conventional U-Net. In this study, we apply the U-Net with several modifications, i.e., employing two loss functions to ensure that the augmented U-Net is well trained (Ibtehaz and Rahman, 2020). The first loss utilizes the distance field of the bone surface as a learned target, where a heatmap-like learned target is generated via Gaussian convolution. As the learning loss reduces, the sigma of the heatmap becomes smaller to enable more accurate bone surface detection. The second loss is to estimate which level of the spine the processed US images belong to, so that the sigma of the aforementioned heatmap can be adjusted (see Figure 4A).

The overall point cloud registration is achieved in a hierarchical coarse-to-fine alignment. The coarse registration step extracts the center of the mass, plus the orientation from the bone surface point clouds detected from the US volume, such that both point clouds between the US and the CT possess the same orientation. This step is performed by decomposing both point clouds via principal component analysis (PCA) and aligning their corresponding principal axes accordingly (Jolliffe and Cadima, 2016). The subsequent fine registration step aligns the specified spine level between the CT vertebrae model and the associated US point clouds. Specifically, the US point clouds near the target spine level are automatically selected for ICP registration (Shi et al., 2006). This step selects and removes neighboring points in an iterative manner, where the criterion is to execute registration for a specified spine level based on the point clouds obtained through different radius configurations, and to eventually select the radius with the smaller MSE loss. After several radius selections, the filtered US point clouds are registered to the corresponding CT spine level model (see Figure 4B).

### Mesoscopic photoacoustic-enhanced drilling probe for local tissue sensing

#### Cancellous bone absorption spectrum

**FIGURE 5:**
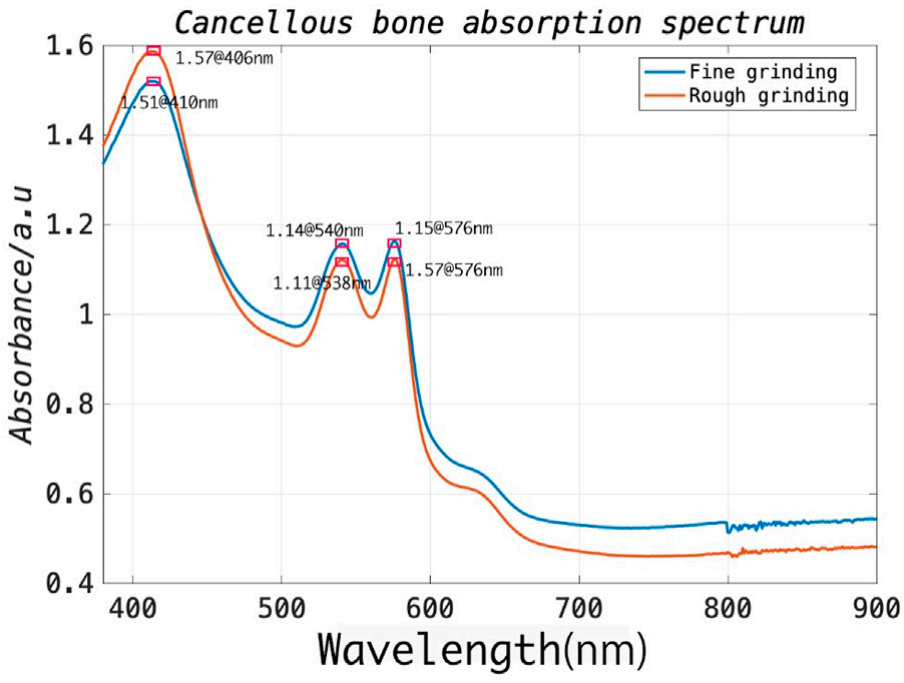
Absorption spectrum of cancellous bone.

In general, during the procedure, the surgical drill is delivered through the muscle and then reaches the outer border of the cortical bone of the lamina. It subsequently further penetrates the cancellous bone of the vertebra through an appropriate entry point on the cortical bone. As the drill advances into the vertebral body, the associated position of the drill within the vertebra provides critical information feedback for achieving the ultimate functional outcome without post-operative complications (Weiss et al., 2010). Since the pedicle structure of the vertebra is highly restricted (a minimum of approximately 3.5 mm), a safe distance between the drill bit and cortical walls (1.44 mm) must be ensured, requiring accurate differentiation between cortical and cancellous bone (An and Benoit, 1998). To this end, the optical absorption characteristics of cortical and cancellous bone should be investigated to select an appropriate laser wavelength for signal excitation. To this end, the spinal model of a bovine sacrificed less than an hour prior was prepared to expose its cancellous bone, and a tweezer was then used to carefully separate the cancellous bone from the cortical bone. The cancellous bone of approximate 2 g was first utilized for rough grinding and placed in the integrating-sphere of the testing machine. The UV spectrophotometer (UV-2600, Shimadzu [Shanghai] Global Laboratory Consumables Co., Ltd.) can cover the wavelength range of 340–960 nm. Subsequently, the coarsely processed cancellous bone was further subjected to fine grinding. The measuring steps were then repeated. As illustrated in Figure 5, the experimental results demonstrated that the strongest absorption exists at 406 nm and stronger absorption peaks at 540 and 576 nm for the prepared bone samples. Therefore, in this study, we prefer a 532 nm pulse laser as the excitation source, which is well suited for spinal bone tissue characterization due to the overall cost considerations associated with the laser source.

#### PA signal acquisition and characterization

**FIGURE 6:**
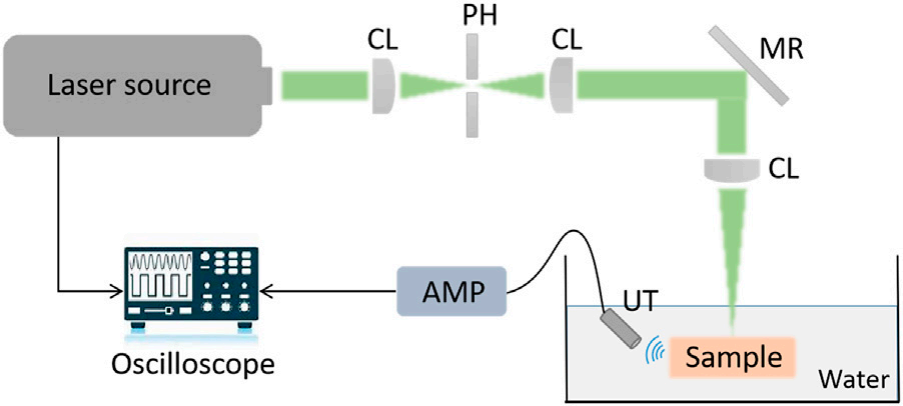
Signal acquisition and characterization of cancellous bone and cortical bone based on a single element ultrasonic transducer.

In this study, we determined an appropriate excitation wavelength as noted in the previous section by analyzing the absorption spectrum of cancellous bone and its cost. With PA imaging, differences of amplitude and frequency between the PA signals on different tissue components have significant impact on the resulting images. It was previously reported that Shubert and Lediju Bell (2018) utilized *ex vivo* photoexcitation and *in vitro* reception to qualitatively characterize cortical and cancellous bone based on PAT. However, for the PAE configuration in this study, PA signal characterization for two tissue components (cortical bone vs*.* cancellous bone) must present a clear distinction. In addition, there is a need to prevent PA signal interference with other tissue components, such as muscle, skin, and blood. To this end, an *ex vivo* measurement experiment using a single element ultrasonic transducer was designed for PA signal characterization of cortical bone and cancellous bone (see Figure 6).

#### PAE-enhanced drilling probe design

**FIGURE 7:**
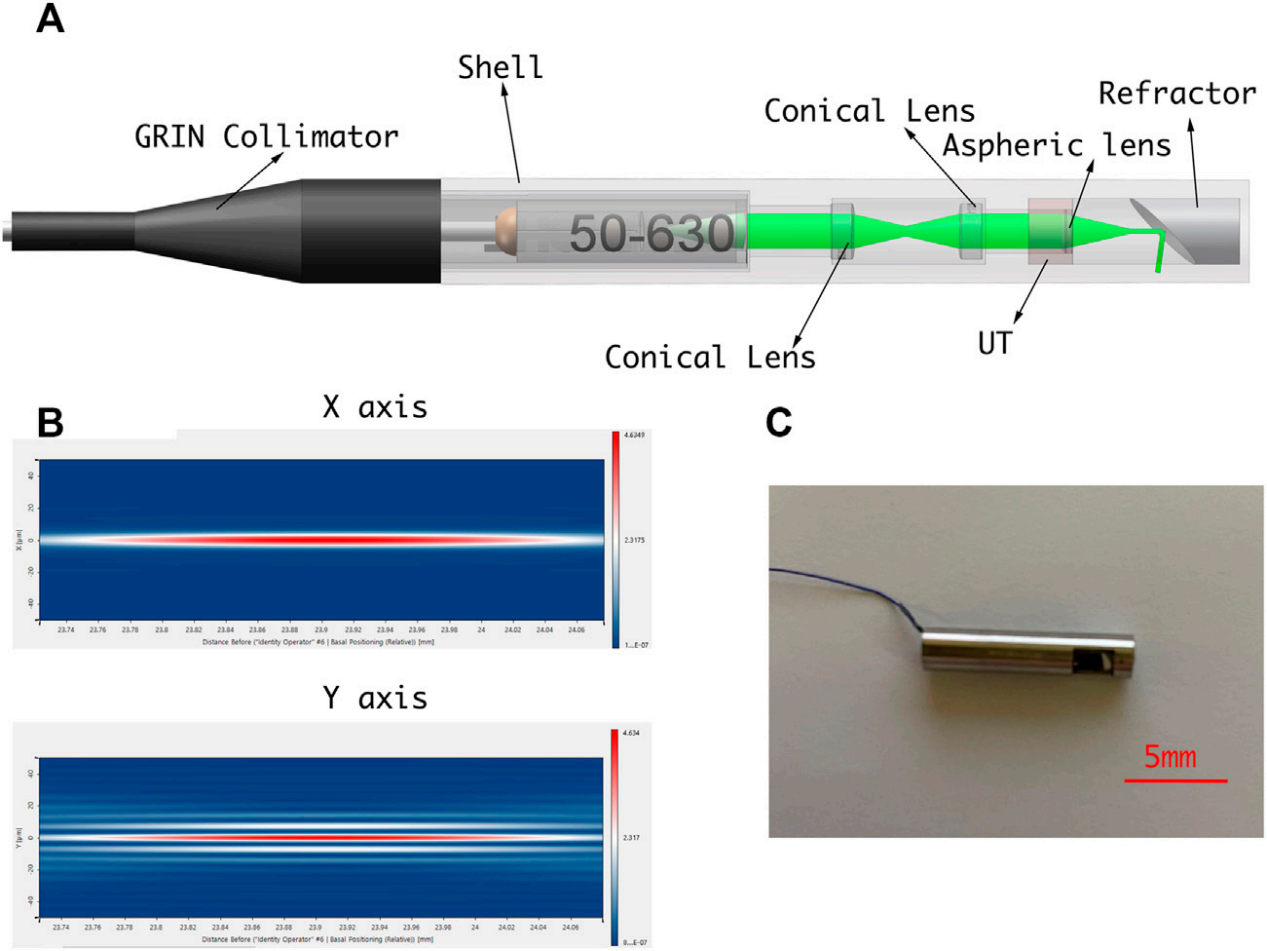
**(A)** Schematic diagram of PAE-enhanced drilling probe. **(B)** Simulation of Bessel beam. **(C)** PAE-enhanced drilling probe prototype.

A PAE-enhanced drilling probe was developed for local tissue sensing inside the vertebrae to facilitate fine orientation adjustment for drilling path optimization. Specifically, a cannulated drill was customized for the integration of a PAE probe based on metal 3D printing. Thus, the developed PAE probe was built into a cannulated drill equipped with a mesopore, allowing for *in situ* PA imaging while perforating the vertebrae. The probe is made up of an optical unit and a single-element ultrasonic transducer. Figure 7A illustrates the optical unit, where, coupled to a single-mode fiber, the laser is collimated into a parallel beam with a diameter of approximately 1 mm, using the GRIN lens collimator (50-630-FC). The parallel beam is guided through the dual cone lenses in opposite directions, and then through the aspherical lens, forming a Bessel beam (Figure 7B). Due to the absence of diffraction, and the ability of the Bessel beam to self-restore after passing through obstacles, it is broadly adopted in measurement, calibration, precision processing, and therapies such as microscopy in particular (Li et al., 2019). After passing through a customized column refractor, the obtained Bessel beam is refracted to the sample surface. The resultant PA signals are also received via refraction from the customized circular probe. The circular single element PA probe is designed into a column to keep its overall dimensions and diameter within an acceptable range. The optical element can be inserted into the inner hole of the ultrasonic probe, which improves its compactness as well as allowing for the coaxiality and confocality of the excitation and receiving signals.

## *Ex vivo* study design

### Sample selection, processing, and fixation

**FIGURE 8:**
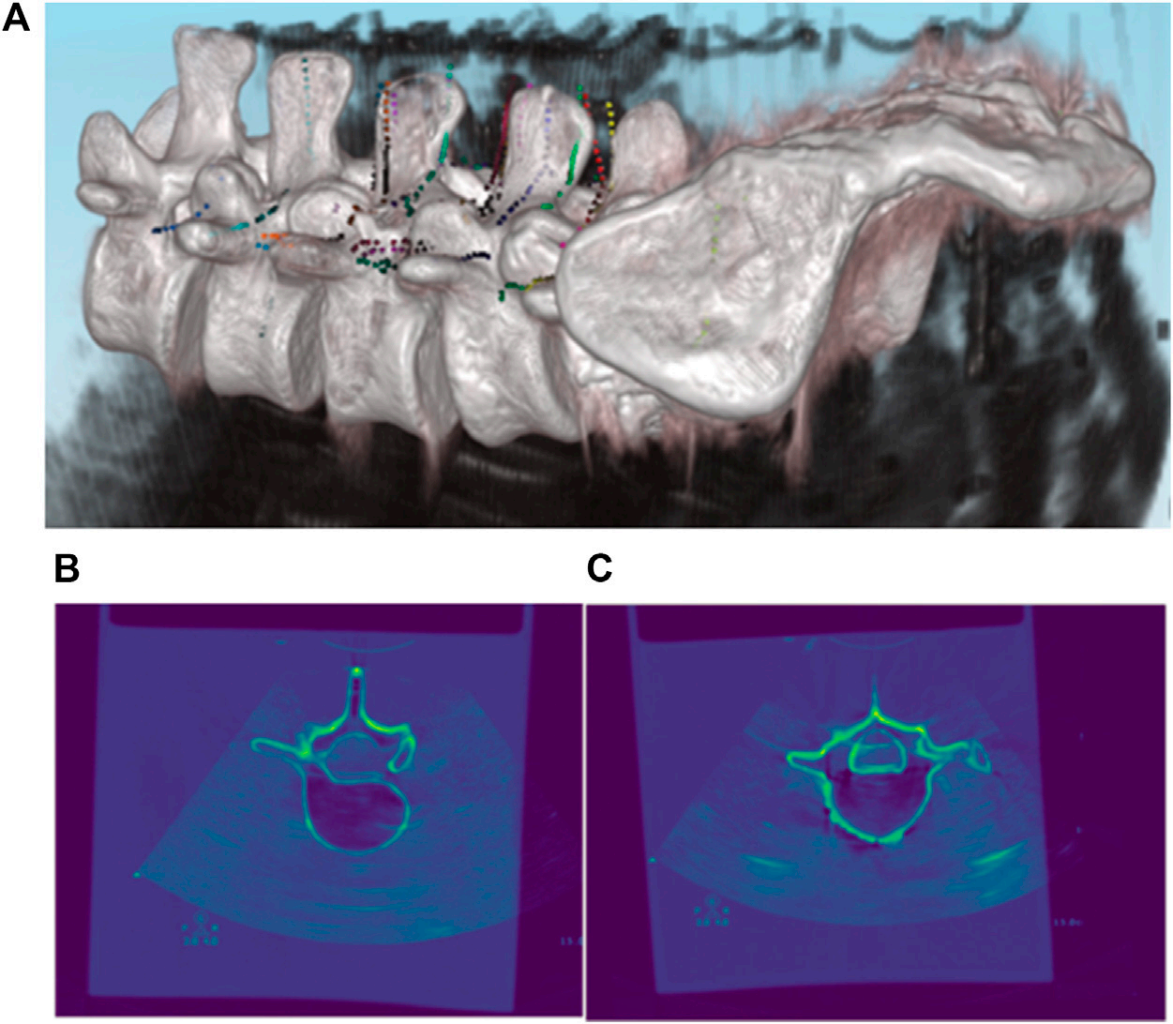
Registration results of pre-operative CT and intra-operative 3D US. **(A)** 3D Rendering of co-registered CT and US images; **(B,C)** 2D Rendering of co-registered CT and US images.

To obtain experimental data for pedicle screw implantation, an intact lumbar vertebra of a bovine was chosen for *ex vivo* studies as the sample comparable to that of a human spine. This is because mammalian spines share similarities in spinal morphology and bone tissue composition. The spine of an adult bovine sacrificed within 3-h was employed for experimental validation. (Age: 3 years, gender: male, weight: 200 kg, vertebra weight: 2.5 kg). Afterward, we used a boning knife to separate the muscle tissue from the spine. The purpose of this step was to make the operation field clearer, and to reduce clutter caused by light shining on the muscle tissue. The intact lumbar vertebra after bone and flesh separation is shown in Figure 8. Afterward, we performed the tissue processing by removing the lumbar vertebra for the subsequent experiments. The entire treated spine was fixed in a transparent acrylic box with glue, and the box filled with water until the tissue was completely covered, in order to significantly reduce attenuation of the PA signal.

### PAE imaging experimental setup

A PAE drilling probe allows for local-scale navigation in a fine-grained manner to facilitate screw implantation more accurately and safely. A torque-enhancement unit and a PAE probe comprise the overall system. Figures 7A,C provide a schematic diagram and system prototype of the probe. After passing through the neutral-density filter (NDF), the pulse laser generated by the Q-switch laser (532 nm, 7 ns, 10 Hz, Xichanye Co., Ltd., China) is divided into two beams by the beam splitter (BS) (the beam ratio is 1:10). The photodiode receives the lower-energy beam, while the other beam is coupled into the optical fiber after being focused by a collimating filter for subsequent PAE imaging. Both signal lines are relatively thin, since the PAE probe is rotated around the main axis of the drill. As shown in Figure 2B, a mechanical torsion increasing unit was designed to prevent the signal wire from breaking during winding with the optical fibers. The outer layer of the optical fiber at the end of the drilling probe, as well as its signal line, are reinforced with a shrinkable heat tube to ensure a relatively stable connection between the outer layer and the signal line. The drill is connected to one end of the heat-shrinkable tube, and the conductive slide ring to the other. The motor’s torque is transmitted to the conductive slide ring via the outer layer during rotation. Simultaneously, the signal line of the single-element ultrasonic transducer relates to the rotor of the conductive slide ring via a fiber that passes through its hollow slide ring and terminates at the end of the optical excitation unit. Since the optical fiber must be rotated as well, a rolling bearing is added at the end, enabling the optical fiber and the probe signal line to be rotated without winding. The maximum laser energy on the sample is approximately 16 mJ/cm^2^, far below the ANSI (American National Standards Institute) safety standard (30 mJ/cm^2^), and the calculation data from UCSB Laser Safety Manual of 19.29 mJ/cm^2^ (UCSB, 2018) (Thomas et al., 2001).

## Results

### Registration of pre-operative CT with intra-operative 3D US

**FIGURE 9:**
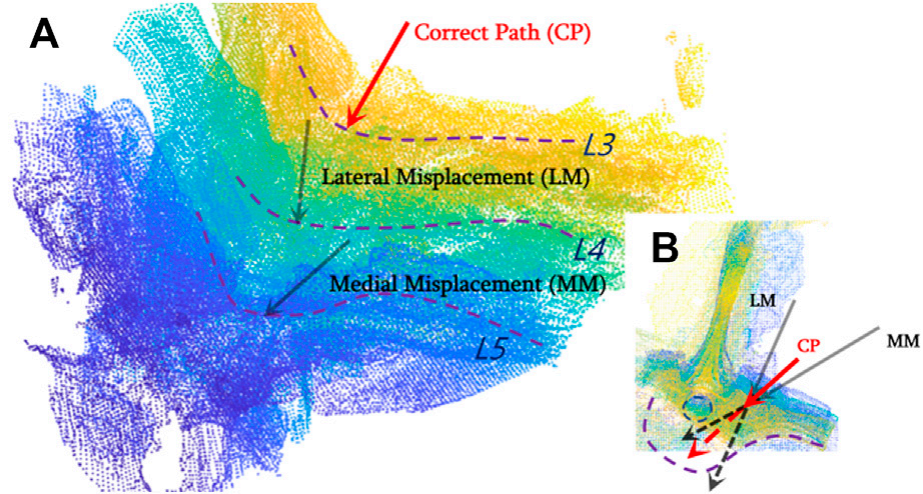
Drilling entry point determination and coarse path planning based on co-registered CT and US images for pedicle screw implantation. **(A)** Three types of screw implantation paths (correct path, lateral misplacement, and medial misplacement) are defined on co-registered CT and US images; **(B)** 2D visualization of three typical screw implantation paths.

As described in the previous section, the most vital task during pedicle screw implantation is determining an appropriate drilling entry and path direction prior to entering the vertebral bone. After the US volume is generated intra-operatively, each vertebral model segmented from pre-operative CT is registered with the point clouds of the bone surface extracted from the US volume, due to the fact that the CT vertebral model can supplement the missing bone structure of the US volume caused by ultrasonic reflection and/or attenuation. As such, it offers a more comprehensive representation of the vertebral bone profile once an inter-vertebral posture variation occurs. As shown in Figure 8, CT data of the lumbar spine are overlain with the spinal sonography in 3D and 2D visualizations. Eventually, based on the co-registered CT and US images, three typical screw implantation paths (i.e., correct path, lateral misplacement, and medial misplacement) are determined (see Figure 9). Along these three coarse paths, the PAE-enhanced drilling probe is advanced for *in situ* bone tissue sensing inside the vertebra and hence for fine path optimization, which is described in the subsequent section.

### PA signal characterization: Cortical bone vs. cancellous bone

**FIGURE 10:**
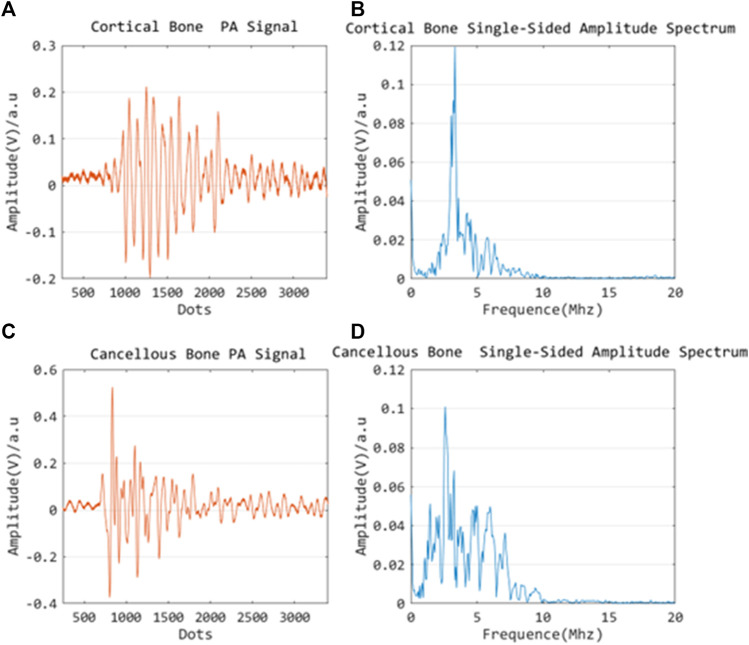
Time domain PA signal characterization of cortical bone **(A)** and cancellous bone **(C)**; Frequency domain PA signal characterization of cortical bone **(B)** and cancellous bone **(D)**.

The PA signals of cortical and cancellous bone are acquired and investigated by the single element ultrasonic acquisition system, as described in the previous section. It is noted that the wavelength and power of the excitation laser are constant, and multiple acquisition results are averaged to reduce crosstalk noise. Since the PA image is a composition of multiple signals with a variety of frequencies in general, the obtained images are transformed to the frequency domain using the Fourier transform for signal characterization. The experimental results are shown in the following figures: Figures 10A,C reflect the signal profile of cortical and cancellous bone in the time domain, while figures 10 B,D demonstrate the signal profile of the cortical and cancellous bone in the frequency domain. Of note, under the same laser energy excitation, the PA signal amplitude peak of cortical bone is slightly lower than that of cancellous bone, and the signals are mostly concentrated in the high-frequency section. The signal response of cancellous bone is slightly stronger than that of cortical bone in terms of amplitude.

### *In situ* PAE sensing via drilling probe

Based on the co-registered CT and US images, three typical screw implantation paths are determined (see Figure 9). Along these three coarse paths, the PAE-enhanced drilling probe advances for *in situ* bone tissue sensing inside the vertebra and hence for fine path optimization.

**FIGURE 11:**
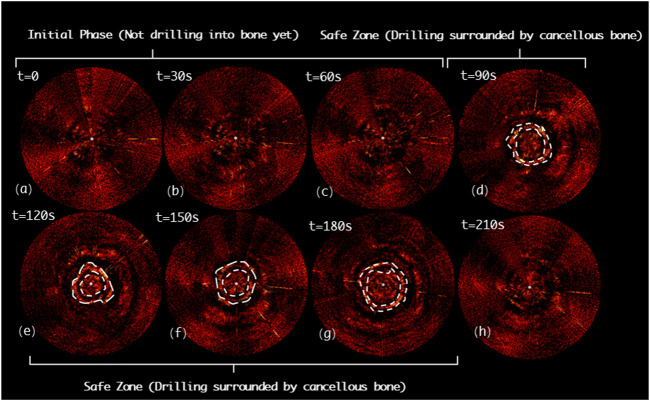
Acquired PAE images along the clinically “correct” path (CP) for *in situ* bone tissue sensing. **(A-C)** Initial phase; **(D-G)** Safe zone; **(H)** Final phase.

Figure 11 indicates the drilling probe insertion along the clinically “correct” implantation path (Correct Path) on the *L3* segment of lumbar spine and demonstrates the acquired PAE images. Such PAE images are obtained at 30*s* intervals. It can be noticed that in the first 90s (see Figures 11A–C), the probe has been delivered into the vertebrae, but has not yet arrived in the pedicle region. During the period of the 90ths~120ths, it can clearly be seen that the probe has penetrated the pedicle region. The PA signals yielded by the surrounding cancellous bone are presented in Figure 11D, and the cancellous bone is distributed evenly around this drilling probe. As the probe is inserted continuously, the PA images obtained (see Figures 11E–G) show the PA signals yielded by the surrounding cancellous bone are still uniformly distributed near the drilling bit. From the 210^th^ *s* on, the PA signals induced by the cancellous bone disappear; at this time the drilling probe has been removed from the vertebra (see Figure 11H).

**FIGURE 12:**
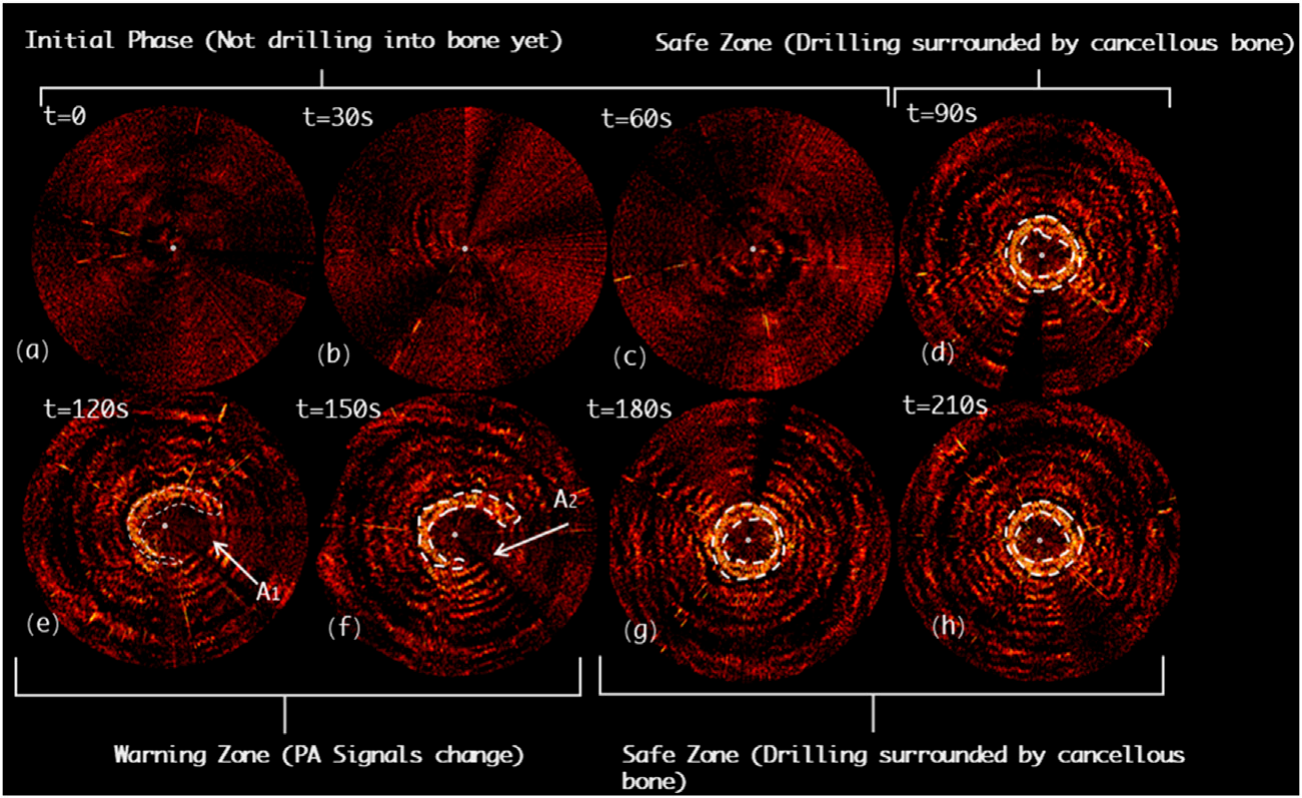
Acquired PAE images along the lateral misplacement path (LMP) for *in situ* bone tissue sensing. **(A-C)** Initial phase; **(D)** Safe zone; **(E-F)** Warning zone; **(G-H)** Safe zone.

Figure 12 shows the drilling probe insertion along the lateral misplacement path (LMP) on the *L4* segment of lumbar spine and demonstrates the acquired PAE images. Such a path often results in lateral cortical breach, thereby bringing potential harm to peripheral vessels or nerves. In the first 90s, the associated PAE images are acquired (see Figures 12A-C), and the drill has not yet been inserted into the pedicle region for bone tissue characterization. During the period of 90ths-120ths, the drilling probe enters the pedicle region. As shown in Figure 12D, the probe is evenly surrounded by cancellous bone in a safe position. During the period of 120ths-180ths, the PA signals change in the regions A_1_ and A_2,_ as highlighted in Figures 12E,F, indicating that new PA signals, different from those of cancellous bone, have been yielded in the regions A_1_ and A_2_. Based on anatomical interpretation, the drill bit is thought to be approaching the interface between the cortical bone and cancellous bone within the pedicle region, indicating the drill bit is in the perforation warning zone. As shown in Figure 12G, starting from the 180^th^ *s*, PA signals like those of the 90^th^ *s* are observed. This reflects that the drill bit remains inside the pedicle region during insertion, only at some sites the drill bit is close to the cortical bone border of the vertebrae, requiring a warning and further correction.

**FIGURE 13:**
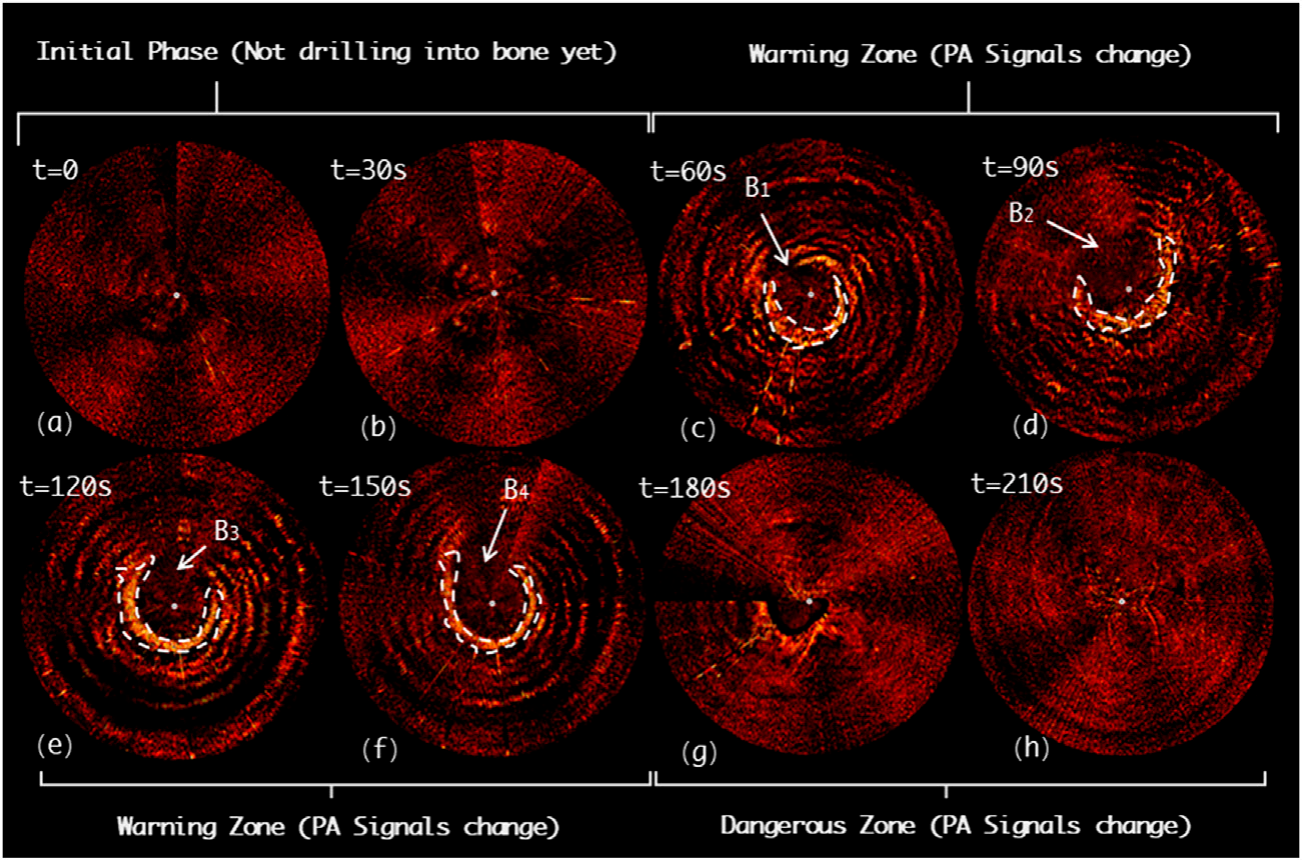
Acquired PAE images along the medial misplacement path (MMP) for *in situ* bone tissue sensing. **(A-B)** Initial phase; **(C-F)** Warning zone; (G-H) Dangerous zone.

Figure 13 demonstrates the drilling probe insertion along the medial misplacement path (MMP) on the *L5* segment of the lumbar spine and demonstrates the acquired PAE images. Such a path frequently results in medial cortical breach, and thereafter causes complications, such as spinal cord injury. In the first 60*s*, the drill has not yet entered the pedicle region (see Figures 13A,B). As shown in Figures 13C-F, starting from the 60^th^ *s*, the variation in the PA signals can be clearly detected (region B_1_ and B_2_), indicating that the drill bit is not surrounded by cancellous bone at this time. Subsequently, from the 120^th^ *s* to 180^th^ *s*, the drill is found to be located inside a perforation warning region (B_3_ and B_4_), where the spinal cord may be at high risk of damage. At 210^th^ *s*, no visible PA signals are detected, revealing that the drill has completely penetrated the medial cortical bone (see Figure 13H).

In order to assess the imaging quality of the PAE images quantitatively, we applied the generalized contrast-to-noise ratio (gCNR) as a metric. The gCNR is a relatively new image quality metric designed to assess the probability of lesion detectability in ultrasound images (Kempski et al., 2020). The gCNR metric is calculated from the overlap of the probability density functions (PDFs) of ROIs inside a target and background. The calculation method can be described in the following formula:

$$OVL=\int_{-\infty }^{\epsilon_{0}}p_{i}\left(x\right)dx+\int_{\epsilon_{0}}^{\infty }p_{o}\left(x\right)dx,$$ (1)

gCNR=1−OVL, (2)

where $p_{i}\left(x\right)$ and $p_{o}\left(x\right)$ are the PDFs for regions inside and outside of a target, respectively, while *OVL* is the total overlap area of the two PDFs derived from photoacoustic images, and is thus the optimal threshold for minimizing the probability of misidentifying background as target and target as background. Table 1 shows the generalized contrast-to-ratio (gCNR) for the acquired PAE images along three typical screw implantation paths (CP, LMP, and MMP).

**TABLE 1:** gCNR of PAE images acquired along three typical screw implantation paths.

	(a)	(b)	(c)	(d)	(e)	(f)	(g)	(h)
CP(Figure 11)	0.13	0.14	0.13	0.37	0.41	0.48	0.53	0.12
LMP(Figure 12)	0.10	0.12	0.19	0.52	0.47	0.49	0.62	0.66
MMP(Figure 13)	0.12	0.13	0.36	0.38	0.42	0.42	0.37	0.15

## Discussion and conclusion

In this study, we developed for the first time a radiation-free and multi-scale navigation and sensing system by applying a PAE-enhanced drilling probe to facilitate pedicle screw implantation optimization. Existing studies either only consider global spatial navigation based on macroscopic anatomical imaging (fluoroscopy or CBCT with radiation; combined US/PAT without radiation) or only take *in situ* tissue sensing, using local sensor-enhanced instruments (acceleration/force sensor; electrical conductivity measurement; EUS probe), into consideration.

The main novelty of this pilot study is that we combine the advantages of global spatial navigation and local tissue sensing techniques. Specifically, the comprehensive navigation system provides global context-awareness from the macroscopic 3D US imaging. 3D US imaging was established for online status updates of the position of the spinal segment to determine an appropriate entry point and coarse drilling path once non-negligible patient motion or relative motion between inter-vertebral segments occurs intra-operatively. The PAE-enhanced drilling probe is subsequently used along the planned coarse insertion path to recognize cortical bone and cancellous bone tissue inside the vertebra near the drill bit for fine orientation correction. To the best of our knowledge, for the first time here, a PAE-enhanced drilling probe is designed, developed, and deployed for pedicle screw implantation in spinal fusion. The *ex vivo* experiment on a bovine spine demonstrates the clinical feasibility of the proposed system, which holds great potential for allowing functionally accurate screw implantation for spine stabilization in complex spine-related interventions. Table 2 demonstrates the advantages and disadvantages of a variety of existing navigation and sensing technologies for pedicle screw implantation. Compared with other state-of-the-art techniques, PA-enabled solutions (either PAT/US macroscopic dual-modal imaging, or PAE-enhanced drilling probe for *in situ* tissue sensing at clinically relevant mesoscopic scale), reveal advantages of non-radiation, high efficacy, moderate deployment complexity, better image quality (gCNR value), and local signal direction information, and hold great potential for facilitating radiation-free and minimally invasive pedicle screw placement.

**TABLE 2:** Comparison of a variety of navigation and sensing techniques for pedicle screw implantation.

Category	Technique	Radiation	Efficacy	Complexity	Image quality (gCNR)	Local signal direction
Global Spatial Navigation	Freehand	√ (High)	Low	Lowest	/	/
	Computer-assisted 2D fluoroscopic navigation	√ (Low)	High	High	/	/
	Computer-assisted CBCT navigation	√ (High)	High	High	/	/
	Computer-assisted CT-fluoroscopic registration	√ (Low)	High	High	/	/
	Macroscopic PAT/US	×	High	Moderate	Highest	/
Local Tissue Sensing	Electrical conductivity measurement	×	Moderate	Low	/	**×**
	Endoscopic US Imaging	×	Moderate	Low	Low	**√**
Multi-Scale Navigation and Sensing	Our proposed solution	×	High	Moderate	High	**√**

However, the challenges of implementing the PAE-enhanced drilling probe for spine stabilization need to be addressed. The size of the PAE-enhanced drilling probe should be more compact, such that its application in clinical practice can meet the need of extremely deformed spine cases, where the pedicle region where the screw is to be implanted is further restricted to 1–2 mm, or even sub-millimeter. Of note, a more compact design scheme based on an optical fiber sensing solution is being investigated to replace the piezoelectrical US transducer.

In addition, the *ns*-pulse laser utilized to generate photoacoustic signals tends to be too expensive compared to other techniques, such as acceleration/force sensors, electrical conductivity measurement probes, and EUS probes. Despite this, the PAE-enhanced drilling probe still holds significant advantages over its counterparts, such as better images, more intuitive visual feedback, and local signal directional information for tissue differentiation and warning. Fortunately, many cost-effective laser sources have become commercially available. It is noted that these could offer a great opportunity to reduce the cost of the PAE-enhanced drilling probe for spinal stabilization in clinical practice.

Since it is difficult to measure the absorption spectrum of solids based on our available measuring instrument, we applied grinding to convert the solid cancellous bone into a powder-like form. We also compared the effect of the degree of grinding on the results of the absorption spectrum, which deviates from the actual absorption spectrum. In addition, the absorption spectrum of solid cortical bone could not be readily tested due to its high density and difficulty in grinding it into powder form. To solve this problem, we measured actual PA signals to distinguish between different tissues. In other words, it elaborates the different absorption resulting from the different composition of solid tissues.

As the morphological characteristics of the cancellous bone *in vivo* are porous and blood-filled, there may be errors due to continuous reflection of the PA signal within the pores during the imaging process. Therefore, our experimental results reveal the relative deviation between the cortical and cancellous bone site in the image at the same test point, and this deviation can help us to differentiate the site. Since the rotation and feed along the axis of the PAE probe is achieved by a surgical drill, the PAE-enhanced straight drill can only accurately serve the function of detecting the safety of the drilled hole. Our future work would be to develop a steerable drilling robot that can avoid dangerous collision situations during surgery.

## Data Availability

The raw data supporting the conclusion of this article will be made available by the authors, without undue reservation.
